# Downregulation of TPX2 impairs the antitumor activity of CD8+ T cells in hepatocellular carcinoma

**DOI:** 10.1038/s41419-022-04645-8

**Published:** 2022-03-10

**Authors:** Xiaochen Wang, Jianchu Wang, Haiyuan Shen, Zongjiang Luo, Xiaojie Lu

**Affiliations:** 1 Department of Hepatobiliary Surgery, Affiliated Hospital of Youjiang Medical University for Nationalities, Baise, Guangxi, 533000 China; 2 Department of Oncology, The First Affiliated Hospital of Anhui Medical University, Hefei, 230022 China

**Keywords:** Cancer, Cell biology, Immunology

## Abstract

Targeting key genes that play dominant roles in T cell dysfunction is an efficient strategy for cancer immunotherapy. Here, we aimed to investigate the role of TPX2 in the antitumor effect of CD8 + T cells in hepatocellular carcinoma (HCC). Flow cytometry was used to assay the level of cell surface markers and cytokines in T cells, through which we found that TPX2 was downregulated in HCC-infiltrating CD8 + T cells. TPX2 depletion restricted the antitumor activity of CD8 + T cells, and TPX2 overexpression increased the antitumor effect of CD8 + T cells in tumor-bearing Cd8^−/−^ mice. TPX2 overexpression improved the antitumor function of human CD8 + T cells and response to anti-PD-1 therapy in an HCC patient-derived xenograft (PDX) mouse model with or without anti-PD-1 therapy. In mechanism, TPX2 promotes the phosphorylation of P65, thus increases the level of p-P65 in nuclear, and p-P65 binds to the promoter of CXCR5, activates its transcription, and increases the level of CXCR5 on CD8 + T cells in a TPX2-dependent way. In conclusion, TPX2 maintains the antitumor effect of CD8 + T cells in HCC by regulating CXCR5 via the NF-κB signaling pathway. Increased TPX2 expression in CD8 + T cells exerts synergistic effects with anti-PD-1 therapy, suggesting a promising immunotherapeutic method in HCC.

## Introduction

Antitumor CD8 + T cell dysfunction is one of the major causes of tumor progression [1, 2]. In the overall cancer-immunity cycle, CD8+ T cells in the peripheral blood can be recruited to the tumor bed by chemotaxis. Subsequently, these CD8+ T cells are activated by diverse tumor-associated antigens, which ideally generates efficiently cytotoxic T cells and finally elimination the tumor [3, 4]. However, in the tumor microenvironment (TME), infiltrated CD8+ T cells under chronic stimulation by diverse tumor-related factors are commonly in a dysfunctional state [2, 5, 6]. These compromised CD8+ T cells are characterized by decreased proliferative potential; increased apoptosis; and the reduced production of effector cytokines, such as TNF-α and IFN-γ, a state also recognized as ‘T cell exhaustion (T_EX_)’. Functionally exhausted CD8+ T cells generally express high levels of inhibitory receptors, such as PD-1, TIM-3, CTLA-4, LAG-3, and TIGIT [7, 8]. Depending on the exposure period to chronic tumor-associated antigens, CD8+ T cells usually progress from full efficiency to partial exhaustion and finally, full exhaustion, accompanied by a gradual reduction in antitumor efficiency and weakened response to therapies targeting immune checkpoint inhibitors (ICIs), such as anti-PD-1 treatment [9, 10]. Recent studies and clinical trials on cancer immunotherapies (e.g., ICIs and CAR-T cells) aimed at improving the antitumor function of CD8+ T cells have shown several encouraging successes with promising outcomes and significantly ameliorated the prognosis of cancer patients [11, 12]. However, some important obstacles need to be overcome, among which TME-induced T cell dysfunction is the most important.

Previous studies have shown that some genes, such as NFAT, BLIMP-1, PSGL1, and TOX, play vital roles in the development of CD8+ T cell dysfunction in the TME [13–17]. Targeting these T_EX_-associated genes can dramatically reverse the altered antitumor efficiency of CD8+ T cells, which indicates that they are promising therapeutic targets for cancer treatment. Nonetheless, the underlying mechanisms of T cell dysfunction in tumors remain largely unclarified.

Hepatocellular carcinoma (HCC) is a well-studied T cell-inflamed cancer with abundant tumor-infiltrating nonefficient antitumor CD8+ T cells, which suggests improving the antitumor function of infiltrated CD8+ T cells is of greater significance in HCC than increasing the infiltration of CD8+ T cells [17]. Previous studies primarily focused on upregulated genes, which usually promote CD8+ T cell exhaustion and suppress tumor progression, and downregulated genes are less well investigated.

In this study, we found that a microtubule nucleation factor (TPX2) is involved in regulating the antitumor activity of CD8+ T cells in HCC. TPX2 was previously studied as a regulator of mitotic spindle formation and shown to act as an inhibitory factor in various cancers, such as lung cancer, renal cancer, colon cancer, and glioma [18–22]. However, the role of TPX2 in antitumor immunity has rarely been studied. Here, we show that TPX2 is downregulated during CD8+ T cell dysfunction and that its absence makes a vital contribution to the development of impaired CD8+ T cell antitumor activity in HCC.

## Results

### TPX2 is downregulated in HCC-infiltrating CD8+ T cells

**Fig. 1:**
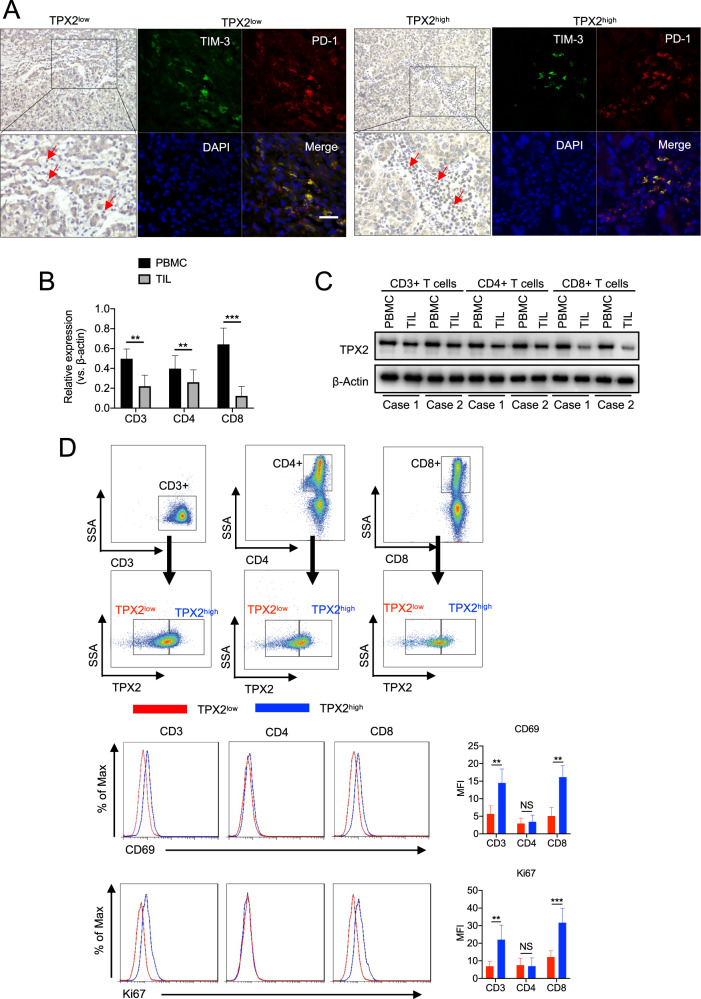
Downregulated TPX2 expression in HCC-infiltrated CD8+ T cells indicated poor antitumor activity. **A** Human HCC samples were divided into two groups (TPX2^low^/TPX2^high^) according to the results of immunohistochemistry (IHC) analysis of TPX2-stained tumor-infiltrating lymphocytes. Immunofluorescent staining of PD-1 and TIM-3 and DAPI staining (for nuclear staining) were performed in the corresponding TPX2^low^ and TPX2^high^ HCC samples. Scale bar: 50 μm. **B** The expression of TPX2 mRNA was detected in CD3+, CD4+, and CD8+ T cells isolated from peripheral blood mononuclear cells (PBMCs) or tumor-infiltrating lymphocytes (TILs). **C** Expression of the TPX2 protein was detected in CD3+, CD4+, and CD8+ T cells isolated from peripheral blood mononuclear cells (PBMCs) or tumor-infiltrating lymphocytes (TILs). **D** Tumor-infiltrating CD3+, CD4+, and CD8+ T cells were divided into the TPX2^low^ and TPX2^high^ groups according to their expression of TPX2 detected by flow cytometry (FC). The activity (CD69) and proliferation (Ki67) of TPX2^low^ and TPX2^high^ CD3+, CD4+, and CD8+ T cells were assayed by FC. The median value of FI (Intensity of fluorescence) was used as the cutoff for the gating strategy for TPX2^high^ and TPX2^low^ groups. **p < 0.01, ***p < 0.001; the two-tailed unpaired Student’s *t*-test was used to compare two groups. PBMCs peripheral blood mononuclear cells, HCC hepatocellular carcinoma, MFI mean fluorescence intensity, TIL tumor-infiltrating lymphocytes. Raw western blot results can be found in the Original data file.

The expression of TPX2 in paraffin slides from 20 HCC samples was detected by IHC. According to the expression levels of TPX2 in tumor-infiltrating immune cells, the 20 HCC samples were divided into two groups: TPX2^high^ and TPX2^low^ (Fig. 1A). Then, the inhibitory receptors PD-1 and TIM-3 were detected by IF, which showed that the proportion of PD-1+TIM-3+ cells was significantly higher in TPX2^low^ HCC tissues (Fig. 1A). These results suggested that tumor-infiltrating immune cells with low TPX2 expression showed a high level of exhaustion. To determine the expression of TPX2 in different subsets of T cells, we separately isolated CD3+, CD4+, and CD8+ T cells from tumor-infiltrating lymphocytes (TILs) and peripheral blood mononuclear cells (PBMCs) from ten HCC patients. We found that compared to those from PBMCs, CD8+ T cells from TILs showed the most dramatic decrease in TPX2 expression (Fig. 1B, C). When we further analyzed the expression of TPX2 in CD3+, CD4+, and CD8+ T cells isolated from TILs by flow cytometry, we observed that CD8+ cells with low TPX2 expression presented the most significant reduction in cell proliferation (Ki67) and activity (CD69) (Fig. 1D). Additionally, we performed an analysis based on the TCGA database, which showed that the expression of TPX2 positively correlated with that of MKI67 in tumor-infiltrating CD8+ T cells in HCC. When we reanalyzed the data from a single-cell sequencing study on HCC, we also found that CD8+ T cells with high TPX2 expression showed a high proliferative ability, as indicated by high MKI67 expression (Supplementary Fig. S1). These results indicated that TPX2 is downregulated in tumor-infiltrating CD8+ T cells in HCC and that low TPX2 expression may predict functional impairment of CD8+ T cells.

### Low TPX2 levels indicate the reduced antitumor effect of CD8+ T cells in HCC

**Fig. 2:**
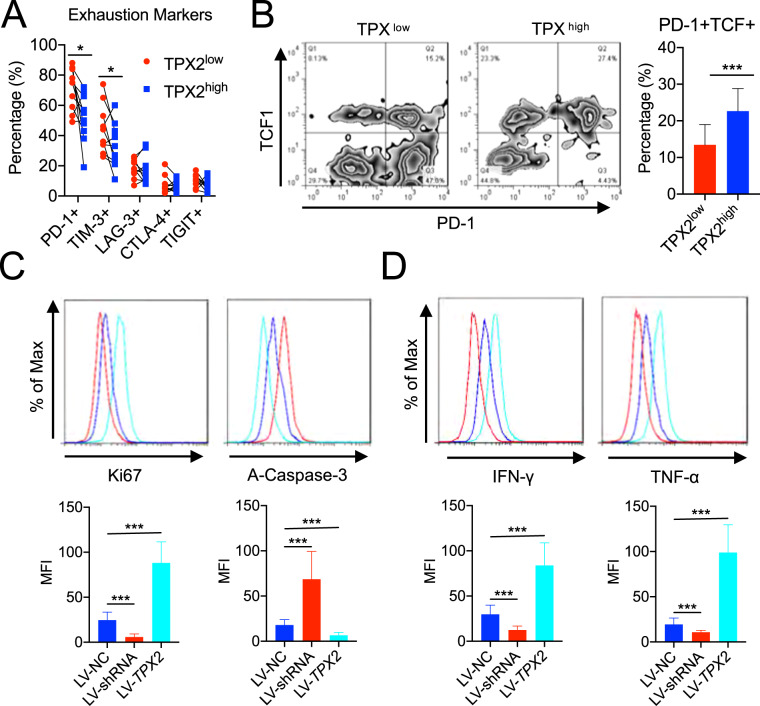
Downregulation of TPX2 in CD8 + T cells indicated low antitumor activity and a poor anti-PD-1 response. **A** The expression of immune checkpoint markers (PD-1, TIM-3, CTLA-4, and TIGHT) was detected in TPX2^low^ and TPX2^high^ (indicated in Fig. 1D) CD8+ T cells by flow cytometry (*n* = 10). **B** The proportion of PD-1 + TCF1+ cells among TPX2^low^ and TPX2^high^ (indicated in Fig. 1D) CD8+ T cells (*n* = 10). **C** Proliferation (Ki67) and apoptosis (A-Caspase-3) were assayed in tumor-infiltrating CD8 + cells in which TPX2 was knocked down (LV-shRNA) or overexpressed (LV-TPX2) (*n* = 6). **D** The production of effector cytokines (IFN-γ and TNF-α) was assayed in tumor-infiltrating CD8+ cells in which TPX2 was knocked down (LV-shRNA) or overexpressed (LV-TPX2) (*n* = 6). *p < 0.05, ***p < 0.001; the two-tailed unpaired Student’s *t*-test was used to compare two groups. A-Caspase-3 activated Caspase 3, IFN-γ interferon gamma, MKI67 Marker of proliferation Ki67, LAG-3 lymphocyte activation gene 3, LV-NC control lentivirus, LV-shRNA lentivirus used to knock down the human TPX2 gene, LV-TPX2 lentivirus used to overexpress the human TPX2 gene, MFI mean fluorescence intensity, PD-1 programmed death 1, TCF1 T cell factor 1, TIGHT T cell immunoreceptor with Ig and ITIM domains, TIM-3 T cell immunoglobulin and mucin domain containing-3, TNF-α tumor necrosis factor alpha.

To investigate the correlation between the expression of inhibitory receptors and TPX2 in CD8+ T cells, we divided TIL-CD8+ T cells into TPX2^high^ and TPX2^low^ subsets (Fig. 1D), after which the expression of PD-1, TIM-3, CTLA-4, LAG-3, and TIGIT was detected by flow cytometry. We observed that PD-1 and TIM-3 were highly expressed in TPX2^low^ CD8+ T cells, which indicated a high exhaustion level (Fig. 2A). The proportions of PD-1+TCF+ cells among TPX2^high^ and TPX2^low^ TIL-CD8+ T cells were also assayed, which showed that the percentage of PD-1+TCF+ cells was significantly higher in TPX2^high^ CD8+ T cells, suggesting a better response to anti-PD-1 therapy (Fig. 2B). When we performed an in vitro exploration with lentivirus to downregulate (LV-shRNA) or overexpress (LV-TPX2) TPX2 in CD8+ T cells isolated from TILs in HCC (Supplementary Fig. S2), we found that TPX2 overexpression increased the proliferation (Ki67) and attenuated the apoptosis (A-Caspase-3) of CD8+ T cells and improved their production of effector cytokines (IFN-γ and TNF-α), while TPX2 downregulation reduced proliferation, enhanced apoptosis, and decreased the production of IFN-γ and TNF-α in CD8+ T cells (Fig. 2C, D). These results suggested that low TPX2 levels indicate the reduced antitumor effect of CD8+ T cells in HCC.

### TPX2 knockdown restricted the antitumor activity of CD8+ T cells

**Fig. 3:**
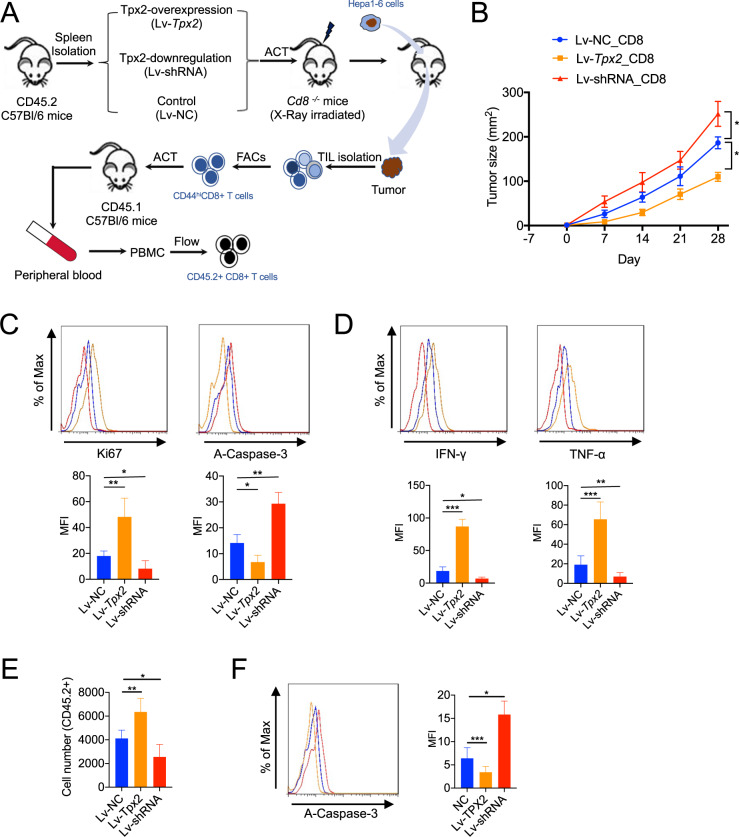
Depletion of TPX2 in CD8+ T cells promoted tumor growth and attenuated the antitumor effect of CD8 + T cells, whereas TPX2 overexpression limited tumor growth and improved the antitumor effect of CD8+ T cells in a mouse HCC model. **A** Illustration of adoptive CD8+ cell transfer in a Cd8^−/−^ tumor-bearing mouse model. CD8 + T cells were isolated from the spleen with anti-CD8 microbeads. LV-shRNA was used for TPX2 knockdown, and LV-Tpx2 was used for TPX2 overexpression in CD8 + T cells. Then, the pretreated CD8+ T cells were transferred into X-ray-irradiated Cd8^−/−^ mice. The mouse HCC cell line Hepa1-6 was used to construct a subcutaneous tumor-bearing model. Thirty days after Hepa1-6 cell inoculation, the tumors were removed for TIL isolation, and CD8+ T cells were isolated with anti-CD8 beads. CD44^hi^CD8 + T cells were sorted by flow cytometry, and a total of 10^4^ CD44^hi^CD8+ T cells were transferred into naïve CD45.1+ transgenic mice (C57BL/6). After 5 days of incubation, the peripheral blood of the recipient mice was collected and used to detect the number and apoptosis of transferred CD45.2+ cells (CD44^hi^CD8+ T cells). **B** Tumor growth was measured weekly after Hepa1-6 cell inoculation (*n* = 8). Day-7 indicates adoptive CD8 + T cell transfer. Day 0 indicates the first day of Hepa1-6 cell inoculation. **C**, **D** Thirty days after Hepa1-6 cell inoculation, tumors were removed for TIL isolation. Proliferation (Ki67), apoptosis (A-Caspase-3) (**C**), and the production of effector cytokines (IFN-γ and TNF-α) (**D**) were assayed in CD8 + T cells from TILs. **E**, **F** The number (**E**) and apoptosis (**F**) of transferred CD45.2+ cells (CD44^hi^CD8 + T cells) were analyzed by flow cytometry. *p < 0.05, **p < 0.01, ***p < 0.001; the two-tailed unpaired Student’s *t*-test was used to compare two groups. A-Caspase-3 activated Caspase 3, IFN-γ interferon gamma, MKI67 Marker of proliferation Ki67, Lv-shRNA lentivirus used to knockdown mouse Tpx2 gene, Lv-Tpx2 lentivirus used to overexpress mouse Tpx2 gene, MFI mean fluorescence intensity, Lv-NC control lentivirus, PBMCs peripheral blood mononuclear cells, TNF-α tumor necrosis factor alpha, TIL tumor-infiltrating lymphocytes.

To explore the role of TPX2 in CD8+ T cell-mediated antitumor immunity, we isolated CD8+ T cells from the spleens of naïve C57BL/6 mice and then used lentivirus to overexpress (Lv-Tpx2) or knockdown (Lv-shRNA) the expression of TPX2 in CD8+ T cells (Supplementary Fig. S3A). These engineered CD8+ T cells were transferred into Cd8^−/−^ C57BL/6 mice, after which mouse HCC cells (Hepa1-6) were subcutaneously inoculated into the mice (Fig. 3A). Tumor growth was observed weekly. Compared to mice that received control CD8+ T cells, mice that received Tpx2-overexpressing CD8+ T cells showed obviously limited tumor growth, while those that received Tpx2-depleted CD8+ T cells showed enhanced tumor growth (Fig. 3B). Tumor-infiltrating CD8+ T cells were then isolated for flow cytometry analysis. We found that Lv-Tpx2-treated CD8+ T cells showed significantly improved proliferation, reduced apoptosis, and increased production of the effector cytokines IFN-γ and TNF-α, while decreased proliferation, increased apoptosis, and the reduced production of IFN-γ and TNF-α were observed in Lv-shRNA-treated CD8+ T cells (Fig. 3C, D).

We next further investigated the proliferative status of the transferred infiltrating CD8+ T cells in HCC. We isolated tumor antigen-activated CD8+ T cells (CD44^high^) (Supplementary Fig. S3B), which were then transferred into naïve CD45.1+ transgenic mice. After 5 days of incubation, the transferred CD8+ T cells (CD45.2+) were isolated for cell counting and flow cytometry analysis of apoptosis. Compared to the control cells, Tpx2-overexpressing CD8+ T cells showed a more proliferative but less apoptotic state, but the Tpx2-depleted CD8+ T cells showed reduced proliferation and increased apoptosis (Fig. 3E, F).

### TPX2 overexpression improved the antitumor function of human CD8+ T cells and the response to anti-PD-1 therapy in a PDX mouse model

**Fig. 4:**
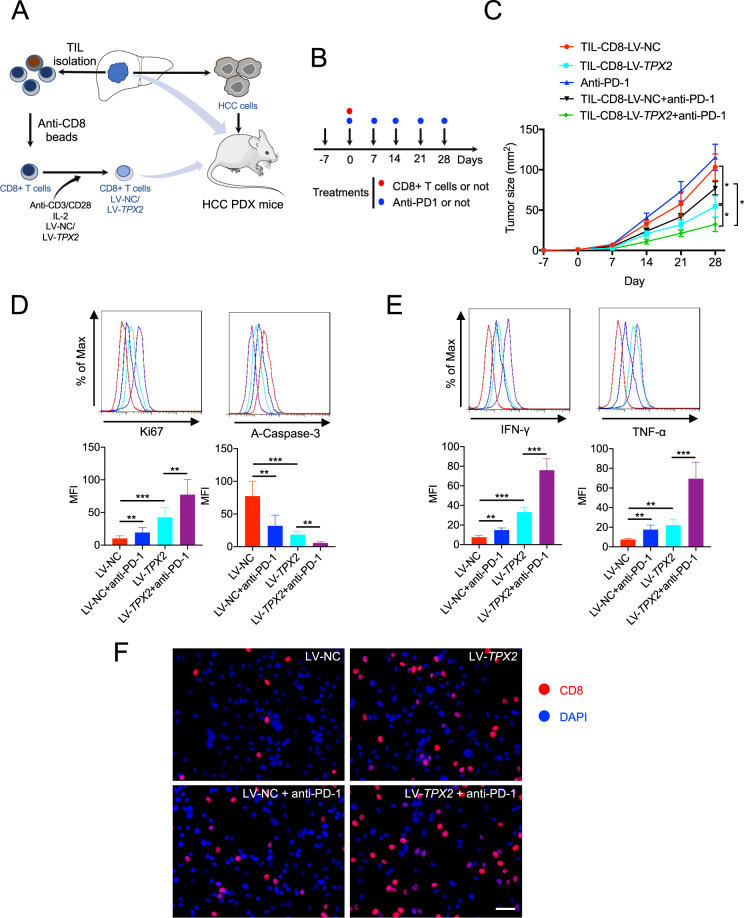
TPX2 overexpression delayed tumor growth and increased the antitumor function and anti-PD-1 response of CD8+ T cells in an HCC PDX mouse model (subcutaneous tumor-bearing model). **A** Illustration of HCC PDX mouse model construction and adoptive T cell transfer for therapeutic purposes. **B** Illustration of CD8 + T cell transfer (i.v. 2 × 10^6^ cells) and/or anti-PD-1 treatment (i.v.) at the indicated time points. Day -7 indicates the first day of human HCC cell inoculation. **C** Tumor growth was observed weekly in human HCC-bearing NSG mice (that received human CD8 + T cells or anti-PD-1 treatment) (*n* = 5). **D**, **E** Thirty days after Hepa1-6 cell inoculation, the tumors were removed for TIL isolation. Proliferation (Ki67), apoptosis (A-Caspase-3) (**D**), and the production of effector cytokines (IFN-γ and TNF-α) (**E**) were measured. **F** CD8 + T cell staining in tumors sections. Scar bar: 50 μm. *p < 0.05, **p < 0.01, ***p < 0.001; the two-tailed unpaired Student’s *t*-test was used to compare two groups. A-Caspase-3 activated Caspase 3, anti-PD-1 antagonist antibody targeting PD-1, HCC hepatocellular carcinoma, IFN-γ interferon gamma, MKI67 Marker of proliferation Ki67, LV-TPX2 lentivirus used to overexpress the human TPX2 gene, MFI mean fluorescence intensity, LV-NC lentivirus used as a control, PDX patient-derived xenograft, TNF-α tumor necrosis factor alpha, TIL tumor-infiltrating lymphocyte.

To investigate whether TPX2 overexpression could improve the antitumor activity of TIL-CD8+ T cells, we isolated CD8+ T cells from fresh HCC tissues, which were then treated with LV-NC as a control or LV-TPX2 for TPX2 overexpression (Supplementary Fig. S4). These cells were subsequently transferred into human HCC PDX mice with or without the administration of anti-PD-1 therapy (Fig. 4A, B). We found that CD8+ T cells overexpressing TPX2 dramatically limited tumor growth, and anti-PD-1 therapy synergistically restricted tumor growth (Fig. 4C). In addition, TPX2-overexpressing CD8+ T cells presented significantly improved proliferation, decreased apoptosis, and increased production of the effector cytokines IFN-γ and TNF-α and infiltration of CD8+ T cells in tumors; these effects were further enhanced upon anti-PD-1 therapy (Fig. 4D, E, F).

**Fig. 5:**
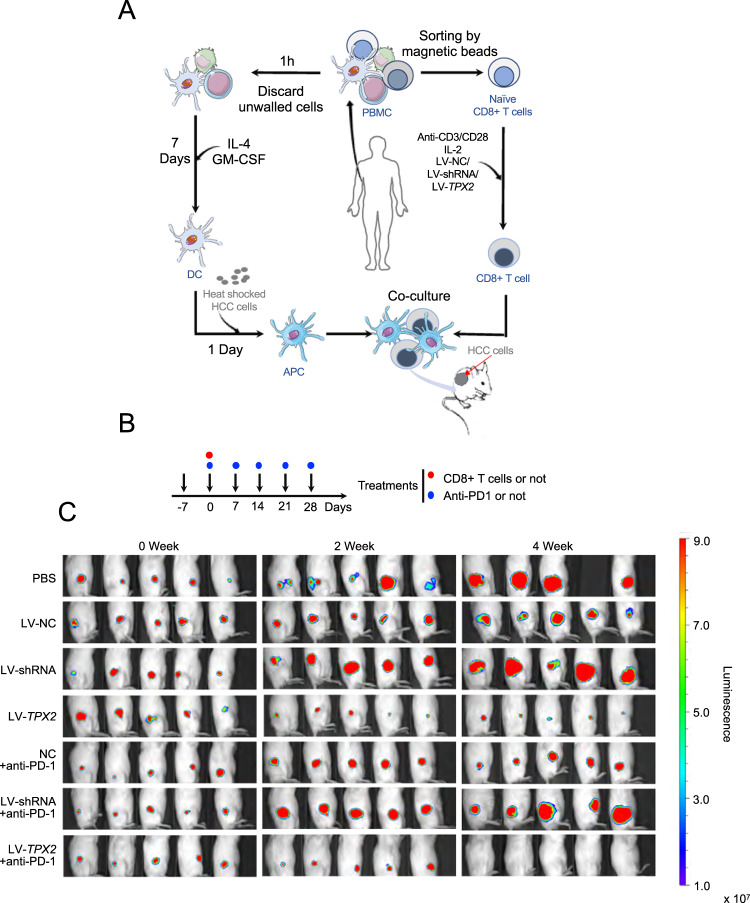
Downregulation of TPX2 in CD8 + T cells promoted tumor growth, whereas TPX2 overexpression delayed tumor growth. **A** Illustration of the generation of HCC-specific CD8 + T cells (see details in the supplementary information) and adoptive CD8 + T cell transfer for the treatment of SMCC-7721-bearing NSG mice (*n* = 5). Naïve CD8 + T cells were isolated from the peripheral blood of healthy donors. **B** Illustration of CD8 + T cell transfer (i.v. 2 × 10^6^ cells) and/or anti-PD-1 treatment (i.v.) at the indicated time points. Day-7 indicates the first day of subcutaneous SMCC-7721 cell inoculation. **C** Tumor growth in mice bearing SMCC-7721 cells was monitored via in vivo bioluminescence imaging with the IVIS imaging system (*n* = 5). anti-PD-1 antagonist antibody targeting PD-1, APC antigen-presenting cell, DC dendritic cell, HCC hepatocellular carcinoma, LV-NC lentivirus used as a control, LV-shRNA lentivirus used to knock down the human TPX2 gene, LV-TPX2 lentivirus used to overexpress the human TPX2 gene, MFI mean fluorescence intensity, PDX patient-derived xenograft, TNF-α tumor necrosis factor alpha, TIL tumor-infiltrating lymphocyte.

We also depleted or overexpressed TPX2 in naïve CD8 + T cells isolated from peripheral blood mononuclear cells (Supplementary Fig. S5A). Naïve CD8 + T cells were incubated with HCC antigen-presenting cells (antigen-loaded dendritic cells, DCs) to generate HCC antigen-specific CD8 + T cells (Supplementary Fig. S5B, C), which were then transferred into HCC tumor-bearing NSG mice for treatment with or without combined anti-PD-1 therapy (Fig. 5A, B). We found that TPX2-overexpressing CD8 + T cells significantly restricted tumor growth, and anti-PD-1 therapy synergistically inhibited tumor growth, while TPX2-depleted CD8 + T cells did not delay tumor growth and showed a poor response to anti-PD-1 therapy (Fig. 5C).

### TPX2 promotes CXCR5 expression in CD8+ T cells by targeting the NF-κB signaling pathway

**Fig. 6:**
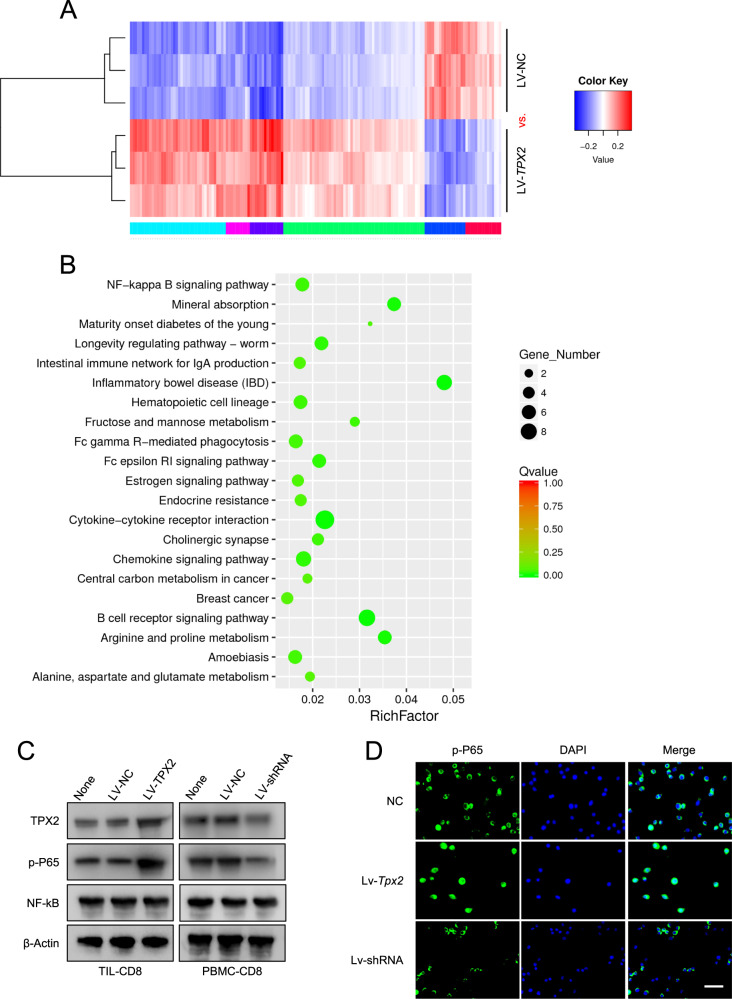
TPX2 promotes the phosphorylation of P65 and activates the NF-κB signaling pathway. **A** Heatmap of the differentially expressed genes induced by TPX2 upregulation (LV-TPX2) in human tumor-infiltrating CD8 + T cells in HCC. **B** KEGG pathway enrichment of the differentially expressed genes induced by TPX2 upregulation. The enriched pathways with *Q*-value < 0.05 (*Q*-value < 0.05 was considered significant) was shown here. **C** The expression levels of TPX2, NF-κB, and p-P65 in LV-TPX2- or LV-shRNA-transfected CD8 + T cells isolated from HCC TILs or PBMCs. **D** p-P65 staining in Lv-Tpx2- or Lv-shRNA-transfected CD8 + T cells. Scale bar: 50 μm. DAPI 4′,6-diamidino-2-phenylindole, for nuclear staining, LV-NC lentivirus used as a control, LV-shRNA lentivirus used to knock down the human TPX2 gene, Lv-shRNA lentivirus used to knock down the mouse Tpx2 gene, Lv-Tpx2 lentivirus used to overexpress the mouse Tpx2 gene, LV-TPX2 lentivirus used to overexpress the human TPX2 gene, PBMCs peripheral blood mononuclear cells, p-P65 phosphorylated P65, TIL tumor-infiltrating lymphocyte. Raw western blot results can be found in the Original data file.

To further understand the underlying mechanism by which TPX2 regulates the antitumor activity of CD8+ T cells, we isolated CD8+ T cells from three fresh HCC tissues, which were divided into two groups; one group was treated with LV-NC as a control and the other was treated with LV-TPX2 for the overexpression of TPX2 (Fig. 6A). These cells were sent for transcriptome sequencing analysis, and differentially expressed genes were selected for pathway enrichment analysis. We found that TPX2-upregulated genes in CD8+ T cells were most significantly enriched in the NF-κB signaling pathway (Fig. 6B). In HCC TIL-CD8+ T cells, TPX2 overexpression dramatically increased the phosphorylation of P65; in human PBMC CD8+ T cells, TPX2 downregulation significantly reduced the phosphorylation of P65, but no change in NF-κB expression was observed (Fig. 6C). In mouse CD8+ T cells isolated from the spleen, the level of p-P65 was increased by TPX2 overexpression and decreased by TPX2 depletion (Fig. 6D).

**Fig. 7:**
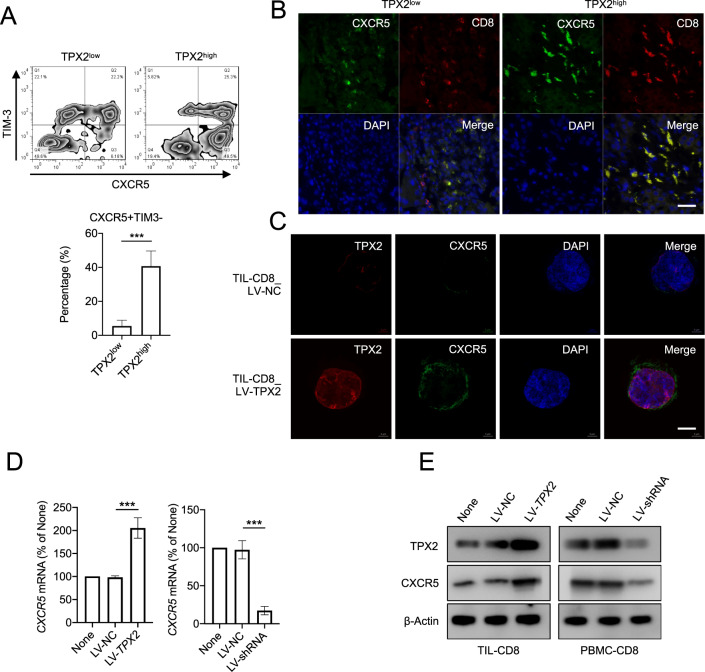
TPX2 regulates CXCR5 expression by promoting P65 phosphorylation. **A** The proportions of CXCR5 + TIM3− cells among TPX2^low^ and TPX2^high^ (indicated in Fig. 1D) tumor-infiltrating CD8+ T cells were analyzed by flow cytometry. **B** IF staining of CXCR5 and CD8 in the corresponding TPX2^low^ and TPX2^high^ HCC samples (indicated in Fig. 1A). Scale bar: 50 μm. **C** IF staining of TPX2 and CXCR5 in tumor-infiltrating CD8+ T cells transfected with LV-TPX2 or LV-NC. Scale bar: 5 μm. **D**, **E** The expression of CXCR5 was detected in tumor-infiltrating CD8+ T cells transfected with LV-TPX2 or LV-NC and PBMC-derived CD8 + T cells transfected with LV-shRNA or LV-NC. ***p < 0.001; the two-tailed unpaired Student’s *t*-test was used to compare two groups. CXCR5 C-X-C chemokine receptor type 5, DAPI 4′,6-diamidino-2-phenylindole, for nuclear staining, LV-NC lentivirus used as a control, LV-shRNA lentivirus used to knock down the human TPX2 gene, LV-TPX2 lentivirus used to overexpress the human TPX2 gene, PBMCs peripheral blood mononuclear cells, p-P65 phosphorylated P65, TIL tumor-infiltrating lymphocyte, TIM-3 T cell immunoglobulin and mucin domain containing-3, TPX2 microtubule nucleation factor. Raw western blot results can be found in the Original data file.

**Fig. 8:**
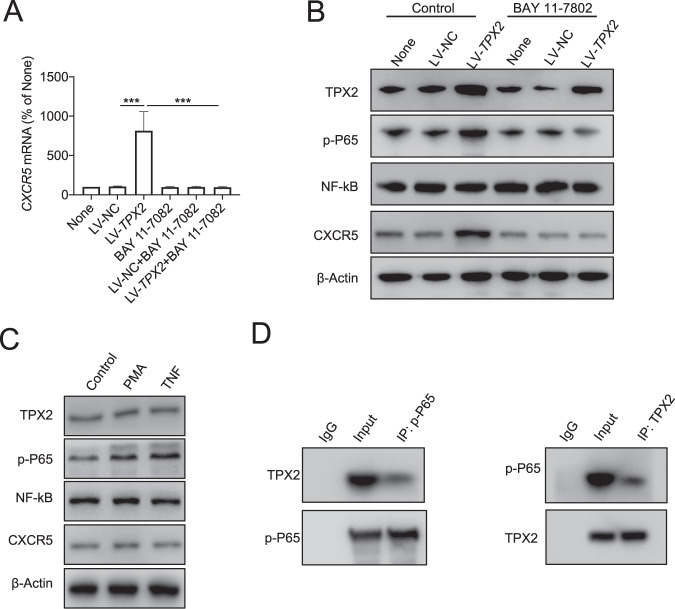
p-P65 regulates CXCR5 expression in a TPX2-dependent way. **A**, **B** The expression of TPX2, CXCR5, NF-κB, and p-P65 was detected in tumor-infiltrating CD8 + T cells transfected with LV-TPX2 or LV-NC and/or treated with an inhibitor of P65 phosphorylation (BAY 11-7802). **C** The expression of TPX2, CXCR5, NF-κB, and p-P65 was detected in tumor-infiltrating CD8+ T cells treated with PMA or TNF. **D** Co-IP (coimmunoprecipitation) analysis of TPX2 and p-P65. ***p < 0.001; the two-tailed unpaired Student’s *t*-test was used to compare two groups. CXCR5 C-X-C chemokine receptor type 5, co-IP coimmunoprecipitation, LV-NC lentivirus used as a control, LV-TPX2 lentivirus used to overexpress the human TPX2 gene, NF-κB nuclear factor kappa-light-chain-enhancer of activated B cells, PBMCs peripheral blood mononuclear cells, p-P65 phosphorylated P65, TIL tumor-infiltrating lymphocyte, TPX2 microtubule nucleation factor. Raw western blot results can be found in the Original data file.

CXCR5 + TIM-3− CD8+ T cells in tumors are polyfunctional cells with high proliferative ability. We found that TIL-CD8 + T cells with high levels of TPX2 also showed a higher proportion of CXCR5 + TIM-3− T cells (Fig. 7A). In HCC tissues with high TPX2 expression, the expression of CXCR5 in CD8+ T cells was also higher (Fig. 7B). In addition, in human TIL-CD8+ T cells and PBMC CD8+ T cells, we found that the expression of CXCR5 was significantly upregulated by TPX2 overexpression and downregulated by TPX2 depletion (Fig. 7C–E). When we treated TPX2-overexpressing cells with BAY 11-7802, an inhibitor of the NF-κB signaling pathway, we observed that the upregulation of CXCR5 induced by TPX2 was abolished (Fig. 8A, B). A previous study reported that p-P65 can bind the promoter of CXCR5 and promote the expression of CXCR5. Interestingly, we found that when we increased the level of p-P65 by PMA or TNF, the expression of TPX2 and CXCR5 was not changed (Fig. 8C and Supplementary Fig. S6). These results indicated that the regulation of CXCR5 is dependent on p-P65 and TPX2. Co-IP analysis with anti-TPX2 or anti-p-P65 antibody showed that TPX2 can bind p-P65 (Fig. 8D). TPX2 is a cytoskeletal protein that plays a vital role in the translocation of cellular components. Therefore, we hypothesized that TPX2 can promote the phosphorylation of P65, thus increasing the level of p-P65 in the nucleus; p-P65 then binds the promoter of CXCR5, activates its transcription, and increases the level of CXCR5 in CD8+ T cells in a TPX2-dependent manner.

## Discussion

In the hepatocellular carcinoma (HCC) tumor microenvironment (TME), persistent inflammation usually recruits a considerable quantity of CD8 + T cells, making HCC a ‘hot’ tumor [23, 24]. However, the antitumor activity of CD8+ T cells is promptly attenuated under stimulation with HCC-associated antigens and other immune inhibitory factors in the TME, a process termed ‘T cell exhaustion’ [1, 7]. During the development of antitumor CD8 + T cell exhaustion, several key genes predominate; these genes may serve as targets for immunotherapies or as prognostic biomarkers for HCC patients [17, 25, 26]. In this study, we showed that TPX2 is downregulated in HCC-infiltrated CD8+ T cells. Although previous studies have shown that TPX2 can promote cancer cell proliferation and act as an unfavorable factor in many cancers, we found that low TPX2 levels in CD8+ T cells indicate compromised antitumor immunity in HCC. In HCC-infiltrated CD8+ T cells, downregulation of TPX2 deactivated NF-κB signaling and decreased the expression of CXCR5, which impaired the antitumor effect of CD8+ T cells. The overexpression of TPX2 in CD8+ T cells improved the antitumor activity of CD8+ T cells and showed a synergistic effect with anti-PD-1 therapy.

Exhausted CD8+ T cells are characterized by low proliferative efficiency and increased apoptotic levels. When we analyzed the single-cell sequencing data reported by Zhang et al. [24], we found that the expression of TPX2 positively correlated with the level of Ki67 in HCC-infiltrated CD8+ T cells, which is consistent with previous studies of TPX2 in cancer cells. When we stratified the HCC tissues into TPX2^high^ and TPX2^low^ groups according to the expression of TPX2 in tumor-infiltrating lymphocytes (TILs), we found a greater proportion of exhausted immune cells (PD-1+TIM-3+) in TPX2^low^ HCC tissues. This suggests that a specific correlation between TPX2 expression and HCC immunity may exist. Considering that T cells are the predominant component of HCC immunity in the TME, we further investigated the expression of TPX2 in CD3+, CD4+, and CD8+ T cells from peripheral blood and TILs. We found that compared to that in PBMCs, the expression of TPX2 in CD8+ T cells from TILs was significantly lower. We also found that CD8+ T cells from TILs with low TPX2 expression showed dramatic decreases in cell activity and proliferative ability. In addition, CD8+ T cells expression low TPX2 showed high levels of inhibitory receptors (PD-1 and TIM3) on the cell surface, indicating a higher exhaustion level. Among current cancer immunotherapies, immune checkpoint blockade (ICB) is the most common. Although ICB has shown great success in the treatment of several cancer patients, it shows poor responses in other patients, even those with high PD-L1 expression [27–29]. TCF1 + PD-1 + CD8+ T cells in tumor tissues were reported to be a valuable predictor of the anti-PD-1 response and associated with better survival outcomes in cancer patients [29–32]. We also found that a higher percentage of the PD-1 + TCF1+ subset among TPX2^high^-expressing HCC-infiltrated CD8 + T cells compared to those expressing low TPX2 levels, which indicated that high TPX2 expression may predict a better anti-PD-1 response.

In vitro analysis of HCC-infiltrated CD8+ T cells showed that the proliferation, activity, and antitumor activity of CD8+ T cells were increased by TPX2 overexpression and decreased by TPX2 knockdown. A bone marrow transfer experiment with TPX2-knockdown or TPX2-overexpressing cells into tumor-bearing CD8^−/−^ mice showed that tumor growth was significantly delayed by TPX2-overexpressing CD8+ T cells but enhanced by TPX2-knockdown CD8+ T cells. Additionally, TPX2-overexpressing tumor antigen-activated CD8+ T cells showed improved viability and attenuated apoptosis. In patient-derived xenograft (PDX) mouse tumor models, we found that TPX2 overexpression increased the antitumor activity of CD8+ T cells, enhanced the response to anti-PD-1 therapy, and dramatically inhibited tumor growth, but TPX2 knockdown promoted tumor growth and compromised the antitumor efficiency of CD8 + T cells.

Mechanistically, we found that TPX2 actively participates in regulating the NF-κB signaling pathway. Transcriptome sequencing analysis showed that TPX2-upregulated genes are significantly enriched in several important immune-related signaling pathways, such as the NF-κB signaling pathway and chemokine signaling pathway. In CD8 + T cells isolated from TILs and PBMCs, we found that TPX2 overexpression promoted the translocation of P65 from the cytoplasm to the nucleus and increased the level of p-P65. CXCR5 + TIM-3-CD8+ T cells are characterized by enhanced self-renewal and multipotency and are largely polyfunctional, which indicates high antitumor activity and a strong anti-PD-1 response [33, 34]. In this study, we found that TPX2 could upregulate the expression of CXCR5 in a p-P65-dependent manner. In combination with the previous finding that P65 can bind the promoter of CXCR5 and promote its transcription [35–37], we hypothesized that TPX2 can promote the phosphorylation of P65, thus increasing the level of p-P65 in the nucleus; p-P65 then binds the promoter of CXCR5 and activates its transcription, increasing the level of CXCR5 in CD8+ T cells in a TPX2-dependent manner. In this study, we found that TPX2 is essential for maintaining the antitumor activity of CD8+ T cells and it is downregulated during the process of CD8+ T cell exhaustion, but the underlying mechanism that contributes to the reduction in TPX2 awaits further investigation. According to the transcriptome sequencing analysis, some other signaling pathways were also significantly enriched, however, we did not fully investigate their potential involvement in TPX2 related function in CD8+ T cells. So, further studies are still needed to explore other molecular mechanisms of TPX2 in regulating CD8+ T cell functions.

Our findings suggest that the upregulation of TPX2 in HCC-infiltrating CD8 + T cells improves the antitumor function of CD8 + T cells and the response to anti-PD-1 therapy through increasing the phosphorylation of P65 and activating the transcription of CXCR5 in CD8 + T cells. However, in our study, the use of the subcutaneous HCC model is a limitation, since it cannot reflect the liver microenvironment. So, more studies with orthotopic HCC models and spontaneous HCC models should be used to further validate the potential of targeting TPX2 to improve immunotherapy efficiency in HCC patients.

## Materials and methods

### Patients and tissue samples

Tumor tissues and peripheral blood were obtained from patients at the Affiliated Hospital of Youjiang Medical University for Nationalities (Guangxi, China). The related clinical information and pathological features are listed in Supplementary Table S1 (for IHC and IF staining), Supplementary Table S2 (for flow cytometry analysis), and Supplementary Table S3 (for transcriptome sequencing analysis). Each patient provided informed consent for tissue analysis before surgery, and the protocol was approved by the Institutional Ethics Committee of the Affiliated Hospital of Youjiang Medical University for Nationalities. All participants signed a written informed consent form.

Other materials and methods in detail are provided in the supplementary materials and methods.

## Supplementary information

Author Contribution Statement

Supplementary Materials and Methods

aj-checklist

Supplementary Figures

Supplementary Table S1

Supplementary Table S2

Supplementary Table S3

Supplementary Table S4

Supplementary Table S5

Information for review_ArrayExpress data

## Data Availability

We declared that all data in this study are available under request. Raw western blot results can be found in the Original data file.
