# Supplementary figures and images for: Downregulation of TPX2 impairs the antitumor activity of CD8+ T cells in hepatocellular carcinoma
Source: Cell Death Dis. 2022 Mar 10;13(3):223. doi: 10.1038/s41419-022-04645-8 (PMC8913637; doi:10.1038/s41419-022-04645-8)

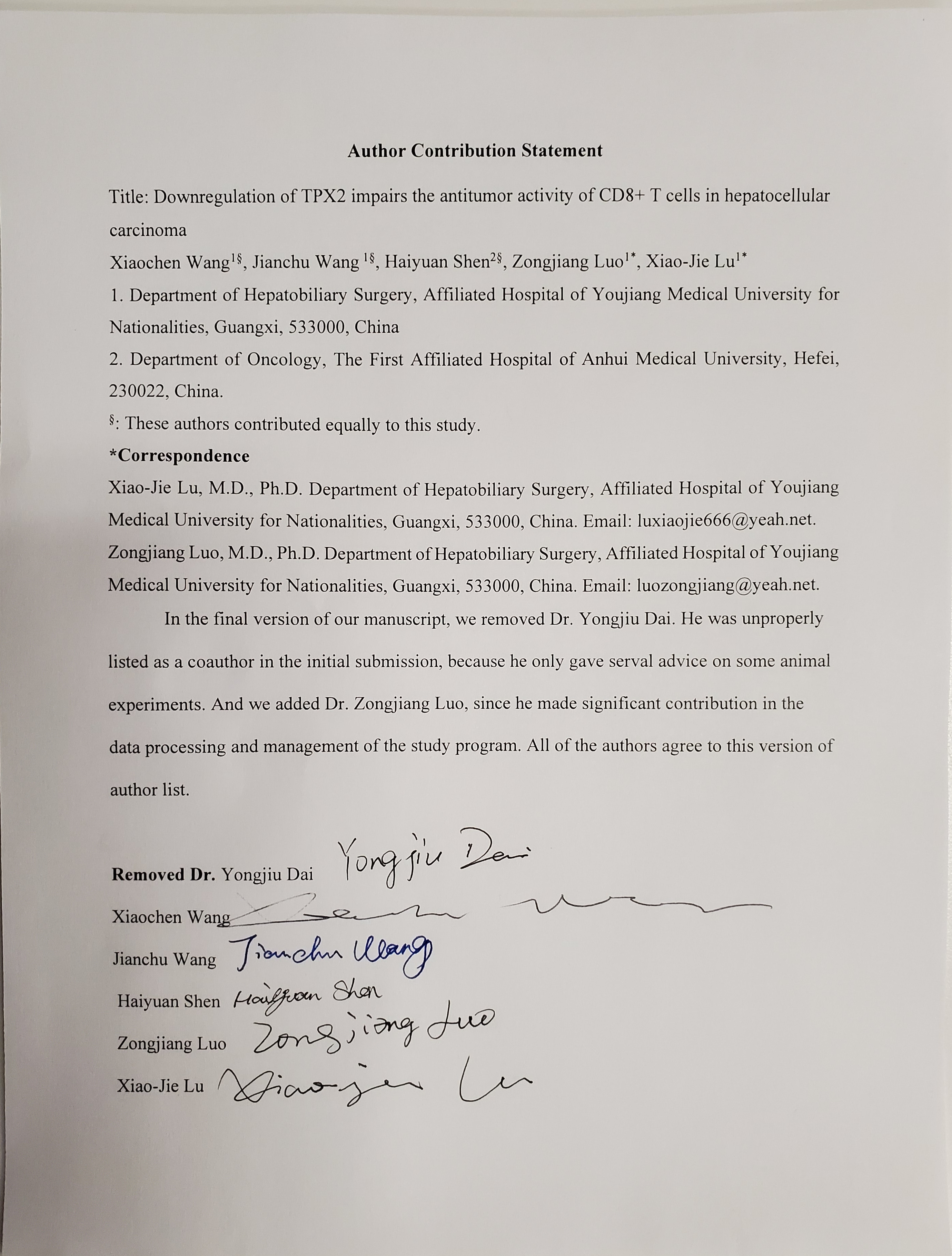

Supplement: Supplementary file 1 — Author Contribution Statement [file 41419_2022_4645_MOESM1_ESM.jpg]
