# Supplementary material for: Downregulation of TPX2 impairs the antitumor activity of CD8+ T cells in hepatocellular carcinoma
Source: Cell Death Dis. 2022 Mar 10;13(3):223. doi: 10.1038/s41419-022-04645-8 (PMC8913637; doi:10.1038/s41419-022-04645-8)
Supplement: Supplementary file 2 — Supplementary Materials and Methods [file 41419_2022_4645_MOESM2_ESM.docx]

**Downregulation of TPX2 impairs the antitumor activity of CD8+ T cells in hepatocellular carcinoma**

Xiaochen Wang^1§^, Jianchu Wang ^1§^, Haiyuan Shen^2§^, Zongjiang Luo^1*^, Xiao-Jie Lu^1*^

1. Department of Hepatobiliary Surgery, Affiliated Hospital of Youjiang Medical University for Nationalities, Guangxi, 533000, China

2. Department of Oncology, The First Affiliated Hospital of Anhui Medical University, Hefei, 230022, China.

^§^: These authors contributed equally to this study.

***Correspondence**

Xiao-Jie Lu, M.D., Ph.D. Department of Hepatobiliary Surgery, Affiliated Hospital of Youjiang Medical University for Nationalities, Guangxi, 533000, China. Email: luxiaojie666@yeah.net

Zongjiang Luo, M.D., Ph.D. Department of Hepatobiliary Surgery, Affiliated Hospital of Youjiang Medical University for Nationalities, Guangxi, 533000, China. Email: [luozongjiang@yeah.net](mailto:luozongjiang@yeah.net).

**Materials and Methods**

**Mice and Cells**

*Cd8*^-/-^ (C57BL/6) mice and CD45.1+ (C57BL/6) mice were obtained from The Jackson Laboratory. NSG (NOD-SCID *IL2rg*^-/-^) mice (BALB/c, male, 6 weeks old) were purchased from Weitong Lihua Experimental Animal Co., Ltd. (Beijing, China). Mice were housed five per cage in individually ventilated cage (IVC) systems in a controlled-temperature room in a specific pathogen-free (SPF)-grade animal facility with a 12-h dark/light cycle at the Animal Center of the Affiliated Hospital of Youjiang Medical University for Nationalities and fed a standard laboratory diet with water. All animal experimental protocols were approved by the Animal Ethics Committee of the Affiliated Hospital of Youjiang Medical University for Nationalities.

The human HCC cell line SMMC-7721 and mouse HCC cell lines Hepa1-6 and HEK-293T were purchased from the Shanghai Institute for Biological Sciences (Shanghai, China). HEK-293T cells were utilized for lentiviral packaging. All cell lines used were authenticated. The cells were cultured in Dulbecco's modified Eagle’s medium (Gibco, CA, USA) supplemented with 10% FBS (Gibco), 100 U/mL penicillin and 100 μg/mL streptomycin (Invitrogen, CA, USA) at 37°C in a humidified incubator with 5% CO_2_.

**T cell isolation**

T cell isolation was performed according to our previous publication [1]. For details, tumor tissues were cut into small pieces and digested with type II collagenase (100 U/ml in HBSS containing calcium and magnesium) and DNase I (20 μg/ml) for 30 min at 37°C, after which a gentleMACS™ Dissociator (Miltenyi Biotec, Bergisch Gladbach, Germany) was used to prepare a single-cell suspension. After centrifugation at 800 ×g at 4°C for 30 min, the T cell fraction was recovered from the interface between the 80% and 40% Percoll (GE Healthcare, Uppsala, Sweden) layers. A FACSAria II cell separator (BD Biosciences, CA, United States) was used for cell sorting with an EasySep^TM^ Mouse CD8 Positive Selection Kit, a CD3 Positive Selection Kit, and a CD4 Positive Selection Kit (STEMCELL, Vancouver, Canada). Ficoll (GE Healthcare) was used to isolate peripheral blood mononuclear cells (PBMCs) and splenocytes, which were purified by negative selection using the EasySep^TM^ Human CD8+ T Cell Enrichment Kit (STEMCELL) and EasySep^TM^ Human Naïve CD8+ T Cell Enrichment Kit (STEMCELL). Cell purity was assessed by flow cytometry, and the purity of all populations was >95%.

**Cell culture and generation of HCC-specific CD8+ T cells**

CD8+ T cells were cultured in RPMI 1640 medium supplemented with 10% fetal bovine serum (Gibco) and stimulated with Dynabeads Human T-Activator CD3/CD28 (Life Technologies, CA, USA), 20 ng/mL human rIL-2, 50 U/mL penicillin, and 50 mg/mL streptomycin for 72 h. The cells were grown in a humidified atmosphere at 37°C with 5% CO_2_.

The isolated PBMCs were cultured in RPMI 1640 medium supplemented with 1% FBS for 1 h, after which adherent monocytes were differentiated into dendritic cells by superinduction with IL-4 (50 ng/ml) and GM-CSF (100 ng/ml) (Invitrogen) and culture for 7 days. The obtained DCs were incubated with heat-shocked HCC cells to obtain antigen-loaded DCs used to generate antigen-presenting cells (APCs). Naïve CD8+ T cells isolated from PBMCs were activated with CD3 and CD28 mAbs as described above and then subjected to lentiviral infection. Then, tumor antigen-specific CD8+ T cells were obtained after the naïve T cells were incubated with APCs for another 3 days.

**Flow cytometry**

Cultured cells were harvested or separated from tumors washed twice with cold PBS, stained with specific antibodies for 30 min at 4 °C in the dark, washed three times with cold PBS, and analyzed by flow cytometry. For intracellular staining, the cells were fixed and permeabilized with a fixation/permeabilization solution kit (BD Biosciences) and 1% paraformaldehyde (PFA) according to the manufacturer’s protocol. For intracellular cytokine detection, the cells were stimulated with 50 ng/mL phorbol myristate acetate (PMA) (Enzo, NY, USA), 1 μg/mL ionomycin (Enzo) and 10 μg/mL brefeldin A (Enzo) for 6 h. Dead cells were excluded using Fixable Viability Stain 620 (BD Biosciences). The cells were then washed tice in PBS, resuspended in FACS washing buffer and analyzed by flow cytometry. The fluorochromes and antibody clones are listed in **Supplementary** **Table S4**. Samples were acquired and recorded in a FACSCanto II flow cytometer (BD Biosciences), and data were analyzed with FlowJo software (TreeStar, Ashland, USA).

**Lentivirus production and transduction**

The *TPX2*-shRNA, *Tpx2*-shRNA, *TPX2*-cDNA, and *Tpx2*-cDNA sequences were manufactured by GenScript Biotechnology (Nanjing, China). Lentivirus was produced via triple transfection of HEK293T cells with a lentiviral transfer vector and the packaging plasmids psPAX2 and pMD2.G at a 0.5:0.375:0.125 ratio using Lipofectamine 3000. The carrier vector used for lentivirus packaging was reconstructed to include the CMV promoter and U6 promoter. Lentivirus was added to 1×10^6^ enriched CD8+ T cells that had been stimulated as described above for 24 h, supplemented with polybrene (10 μg/ml, Sigma-Aldrich, MO, USA) and centrifuged at 2000 rpm and 32°C for 2 h. The targeting sequences of the siRNAs against human and mouse *TPX2* are listed in **Supplementary** **Table S5**.

**In vivo tumor model and treatment**

Xenograft HCC model mice were constructed as described in our previous publication. For details, 2×10^6^ patient-derived primary cancer cells (for the PDX HCC model) or 2×10^6^ SMCC-7721 cells (transfected with lentivirus for overexpression of the luciferase gene) were mixed with Matrigel 1:7 in a total volume of 100 µL and subcutaneously inoculated in the right groin of NSG mice (BALB/c, male, 6 weeks old). LV-*TPX2*-treated or control human TIL-CD8+ cells (2×10^6^ cells/mouse) or *TPX2*-overexpressing, *TPX2*-knockdown or control lentivirus-treated APC-stimulated naïve CD8+ T cells (1×10^7^ cells/mouse) were injected into the caudal vein for therapy. A human anti-PD-1 antibody (nivolumab) (10 μg/kg) or an IgG control was injected into the mice i.p. once per week for the indicated duration. Tumor growth in mice bearing SMCC-7721 cells was monitored via in vivo bioluminescence imaging with the IVIS imaging system using Living Image acquisition and analysis software (Caliper Life Sciences, Mass, USA).

CD8+ T cells were isolated from the spleens of 6-week-old male C57BL/6 (CD45.2) mice, transferred to *Cd8^-/-^* mice (male, 6 weeks old), and the mice were then immediately inoculated with Hepa1-6 cells. On the 30th day after the tumor cells had been implanted, CD44^hi^CD8+ T cells (10^4^) were isolated from the tumors in *Cd8^-/-^* mice and transferred to naïve C57BL/6 (CD45.1) mice (male, 6 weeks old). The blood of the recipient mice was collected to isolate peripheral blood mononuclear cells and for subsequent analysis.

**RNA sequencing**

Total RNA was extracted from LV-*TPX2*-treated TIL-CD8+ T cells or control cells by TRIzol reagent (Life Technologies) and then purified with DNase I (Qiagen, Dusseldorf, Germany). Library preparation, quality control and sequencing were performed by Genewiz (Suzhou, China). For details, the RNA-seq library was constructed from the RNA samples according to a strand-specific RNA sequencing library preparation protocol. Sequencing was performed on an Illumina MiSeq with paired 50 bp reads using the MiSeq Regent Kit v3 (Illumina, CA, USA). Reads were aligned to mm10 using TopHat and assembled into transcripts using Cufflinks. The FPKM value was used for the analysis for differently expressed genes between LV-*TPX2*-treated TIL-CD8+ T cells and control cells. KEGG pathway enrichment was performed to explore the TPX2 related pathways in CD8+ T cells. Raw data were deposited in the ArrayExpress database (E-MTAB-10040).

**Immunoblotting and immunofluorescence**

Cells and liver samples were homogenized and ruptured using ice-cold RIPA buffer containing fresh protease and phosphatase inhibitors (Beyotime, Nantong, China), and the protein concentration was measured using the BCA assay (Beyotime). After denaturation, protein samples were subjected to SDS-PAGE, followed by immunoblotting with the antibodies listed in Table S4. Blots were visualized with an enhanced chemiluminescence detection kit (Pierce, Thermo Scientific, USA) and the ChemiDoc^TM^ MP Imaging System (Bio-Rad).

Cells were fixed with 4% paraformaldehyde and incubated with primary antibody at 4°C overnight. After washing with PBS, cells were incubated with fluorescence-conjugated secondary antibody (Invitrogen) and briefly stained with DAPI (Life Technology). Images were captured using an inverted confocal fluorescence microscope.

**Quantitative real-time PCR**

Total RNA was extracted using TRIzol reagent (Invitrogen), and complementary DNA (cDNA) was synthesized using a reverse transcription PCR kit (Roche) according to the manufacturer’s instructions. qRT-PCR was performed with Maxima SYBR Green qPCR Master Mix (Applied Biosystems) on an ABI Prism 7900HT (Applied Biosystems, Foster City, CA, USA). The sequences of the primers used are listed in Table S5.

**Statistical analysis**

All of the experiments in this study were repeated for at least three times with no less than 3 samples in each experiment for the generation of mean value. No samples were excluded during the analysis. For animal studies, no specific methods were used for the selection of the mice used for our research, and no blinding was done. Data are presented as the mean ± SD or ± SEM as indicated in the figure legends. Differences in means were analyzed by Student’s *t*-test and one-way ANOVA. The log rank test was performed for analysis of Kaplan-Meier curves. Differences with *p* values < 0.05 were considered significant (*: *p* < 0.05, **: *p* < 0.01, ***: *p* < 0.001).

**References**

1. Wang X, He Q, Shen H, Xia A, Tian W, Yu W, Sun B: **TOX promotes the exhaustion of antitumor CD8(+) T cells by preventing PD1 degradation in hepatocellular carcinoma**. *J Hepatol* 2019, **71**(4):731-741.
