# Supplementary material for: Downregulation of TPX2 impairs the antitumor activity of CD8+ T cells in hepatocellular carcinoma
Source: Cell Death Dis. 2022 Mar 10;13(3):223. doi: 10.1038/s41419-022-04645-8 (PMC8913637; doi:10.1038/s41419-022-04645-8)
Supplement: Supplementary file 4 — Supplementary Figures [file 41419_2022_4645_MOESM4_ESM.docx]

**Supplementary Figures**

**
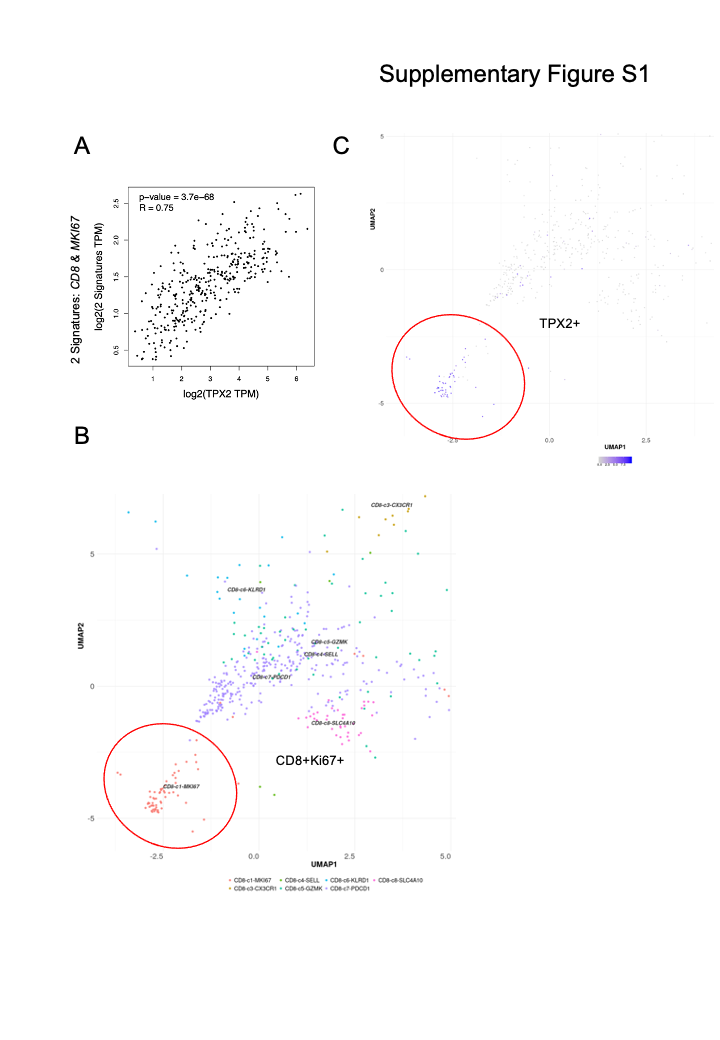
**

**Supplementary Figure S1.** **TPX2 was highly expressed in tumor-infiltrating CD8+ T cells with a high proliferative ability.** (A) The expression level of *TPX2* positively correlated with that of *MKI67* in CD8+ T cells. Data analysis was performed via the online software <http://gepia.cancer-pku.cn>. (B, C) In the subset of CD8+MKI67+ cells, the expression of *TPX2* was significantly higher than that in other subsets of tumor-infiltrating CD8+ T cells in HCC. Data analysis was performed via the online software <http://cancer-pku.cn:3838/HCC/>.


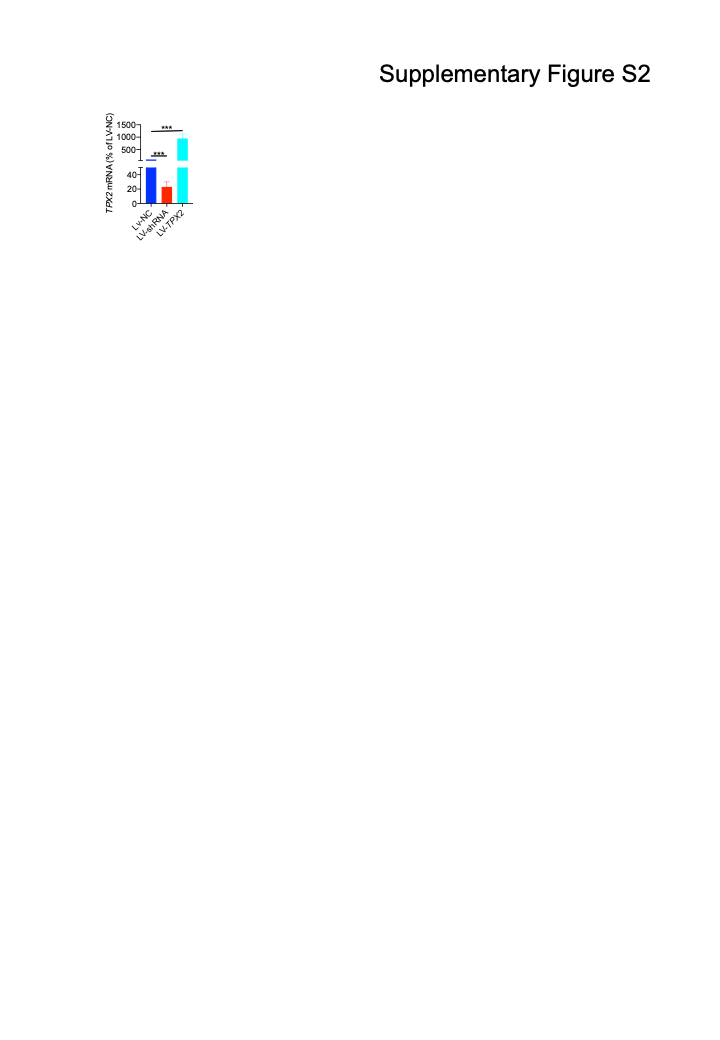


**Supplementary Figure S2. The expression of TPX2 in tumor-infiltrating CD8+ cells.** The expression of TPX2 was detected in tumor-infiltrating CD8+ cells in which TPX2 was knocked down (LV-shRNA) or overexpressed (LV-*TPX2*) (n=6). ***p <0.001; the two-tailed unpaired Student’s t-test was used to compare 2 groups. LV-NC, control lentivirus; LV-shRNA, lentivirus used to knock down the human *TPX2* gene; LV-*TPX2*, lentivirus used to overexpress the human *TPX2* gene; MFI, mean fluorescence intensity.


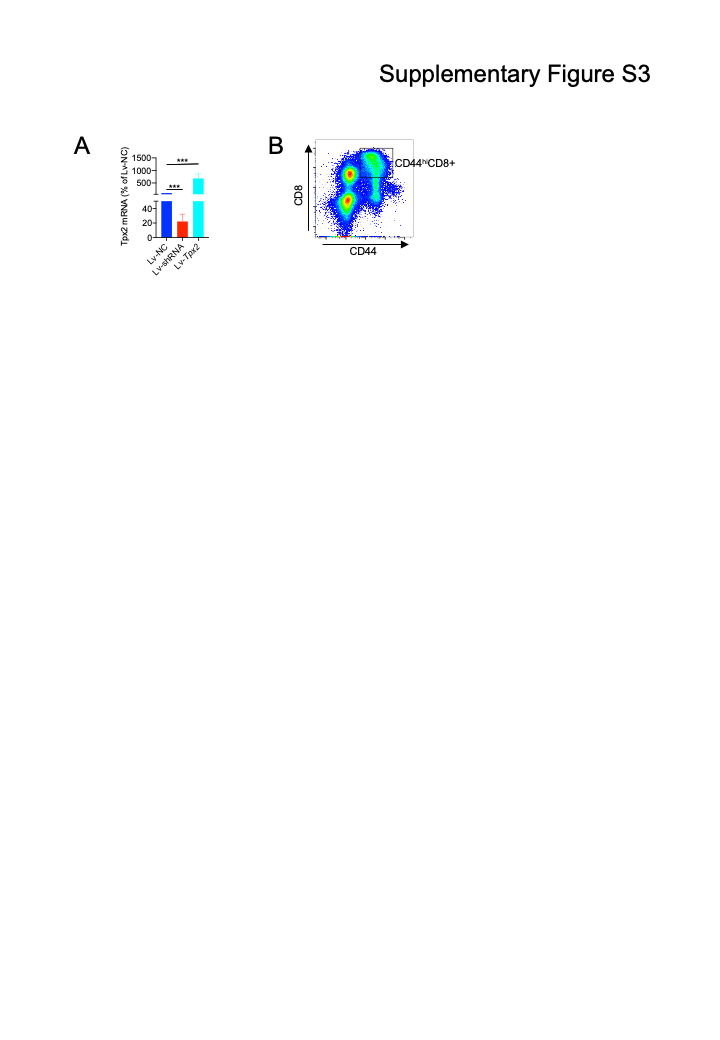


**Supplementary Figure S3. The expression of TPX2 in tumor-infiltrating CD8+ cells; gatingstrategy for CD44^hi^CD8+ T cells. (A)** The expression of TPX2 was detected in tumor-infiltrating CD8+ cells in which TPX2 was knocked down (LV-shRNA) or overexpressed (LV-*TPX2*). (B) CD44^hi^CD8+ T cells were sorted by flow cytometry. ***p <0.001; the two-tailed unpaired Student’s t-test was used to compare 2 groups. Lv-NC, control lentivirus; Lv-shRNA, lentivirus used to knock down the mouse *Tpx2* gene; Lv-*Tpx2*, lentivirus used to overexpress the mouse *Tpx2* gene.


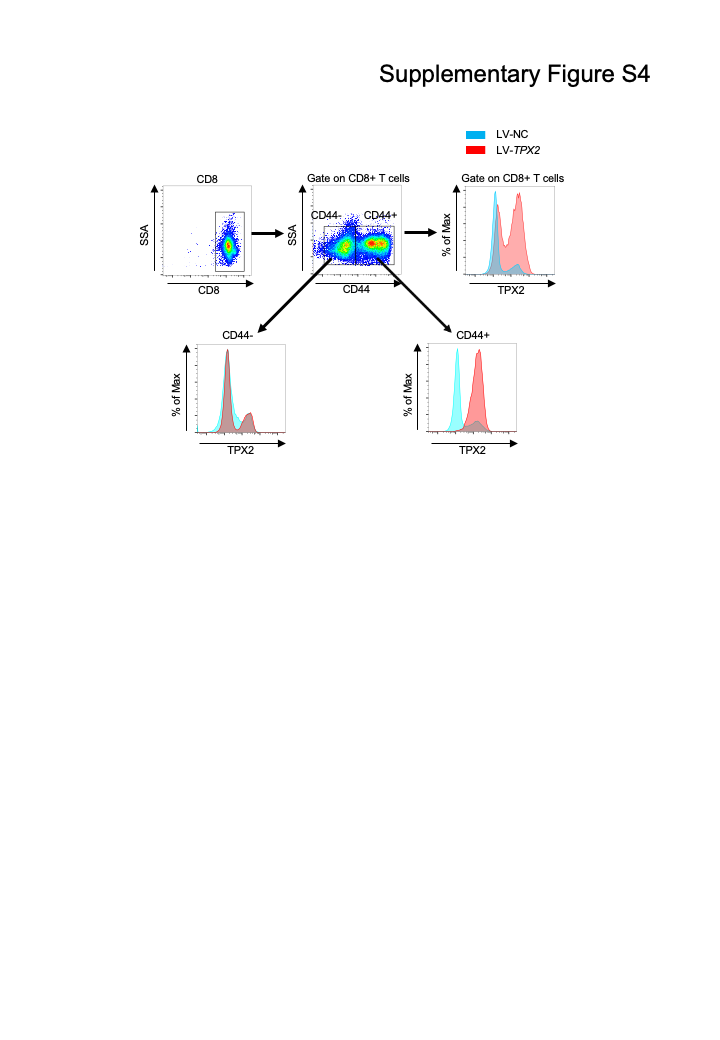


**Supplementary Figure S4. Overexpression of TPX2 in CD8+ T cells.** (A) In CD8+ T cells isolated from tumor-infiltrating lymphocytes, LV-*TPX2* induced the upregulation of TPX2 in mostly CD44+CD8+ T cells. LV-*TPX2*, lentivirus used to overexpress the human *TPX2* gene; LV-NC, lentivirus used as a control.


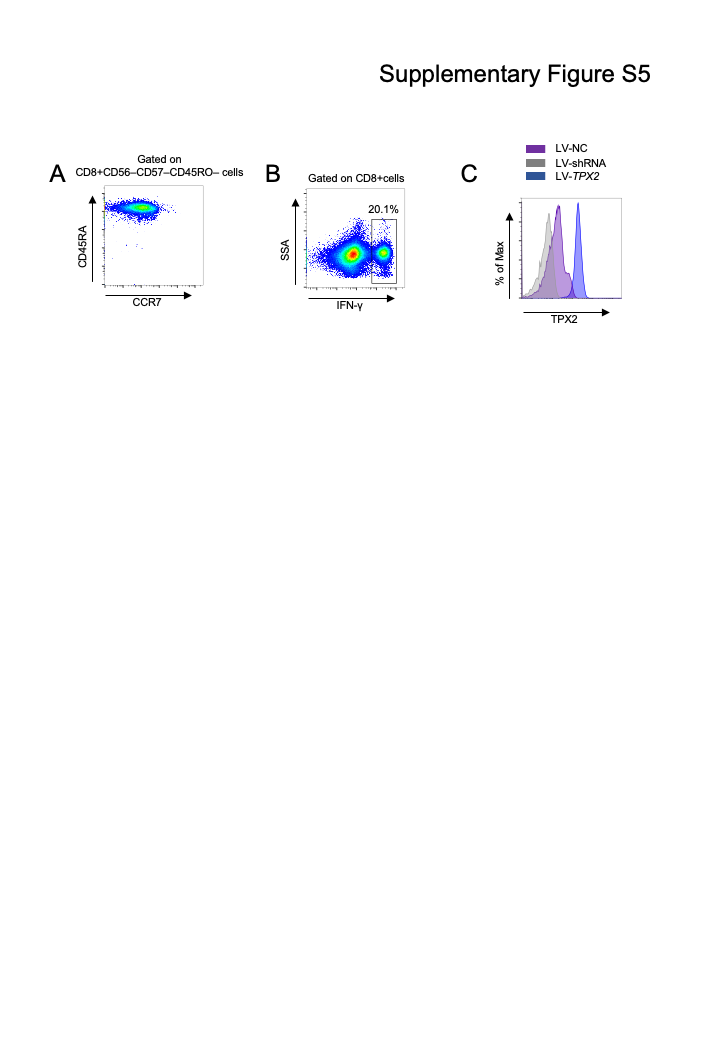


**Supplementary Figure S5. TPX2 overexpression and TPX2 downregulation in CD8+ T cells.** (A) Gating strategy used to sort naïve CD8+ T cells by flow cytometry. (B) The efficiency of CD8+ T cell activation by viable HCC cells. (C) The expression of TPX2 was detected in CD8+ cells in which TPX2 was knocked down (LV-shRNA) or overexpressed (LV-*TPX2*) by flow cytometry. IFN-γ, interferon gamma; LV-NC, lentivirus used as a control; LV-shRNA, lentivirus used to knock down the human *TPX2* gene; LV-*TPX2*, lentivirus used to overexpress the human *TPX2* gene; TIL, tumor-infiltrating lymphocyte.


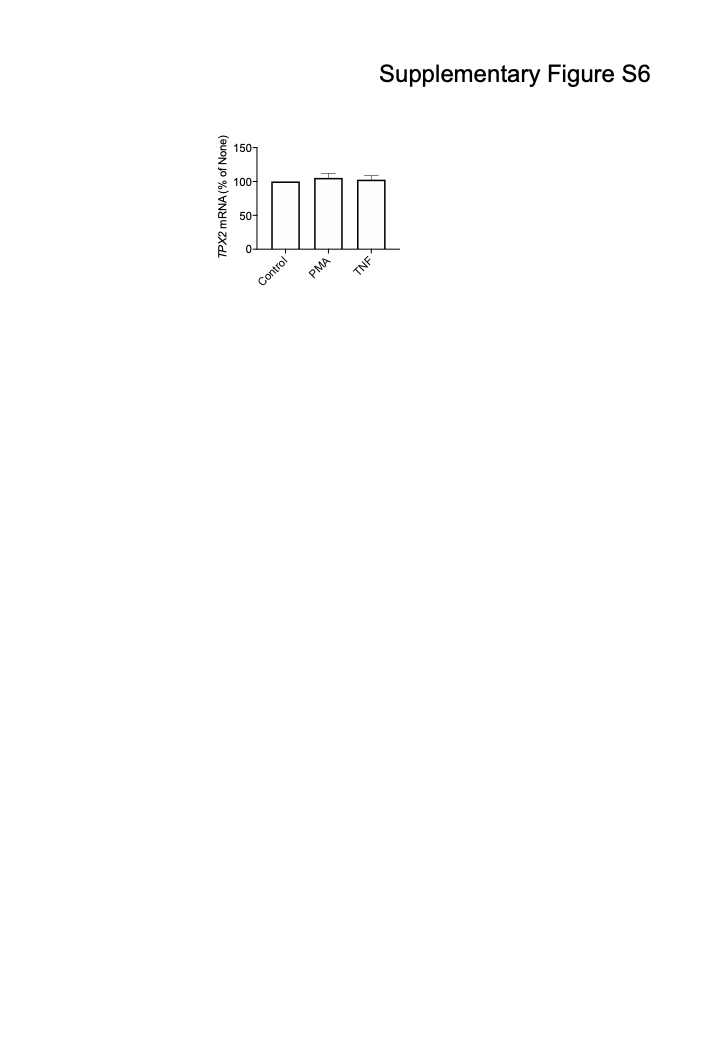


**Supplementary Figure S6. The expression of TPX2, CXCR5, NF-κB and p-P65 was detected in tumor-infiltrating CD8+ T cells treated with PMA or TNF.** CXCR5, C-X-C chemokine receptor type 5; NF-κB, nuclear factor kappa-light-chain-enhancer of activated B cells; p-P65, phosphorylated P65; TIL, tumor-infiltrating lymphocyte; TIM-3, T-cell immunoglobulin and mucin-domain containing-3, TPX2, microtubule nucleation factor.
