# Supplementary material for: Downregulation of TPX2 impairs the antitumor activity of CD8+ T cells in hepatocellular carcinoma
Source: Cell Death Dis. 2022 Mar 10;13(3):223. doi: 10.1038/s41419-022-04645-8 (PMC8913637; doi:10.1038/s41419-022-04645-8)
Supplement: Supplementary file 6 — Supplementary Table S2 [file 41419_2022_4645_MOESM6_ESM.docx]

**Supplementary Table S2.** Patient cohort for flow cytometry analysis.

| Feather | Number |
| --- | --- |
| All cases | 30 |
| HBV |  |
| Yes | 18 |
| No | 12 |
| Age(years) |  |
| <60 | 8 |
| ≥ 60 | 22 |
| Gender |  |
| Male | 24 |
| Female | 6 |
| Tumor size (cm) |  |
| < 3 | 6 |
| 3-5 | 17 |
| > 5 | 7 |
| Differentiation grade |  |
| Well | 6 |
| Moderate | 18 |
| Poorly | 6 |
| Tumor capsular |  |
| Incomplete | 4 |
| Complete | 26 |
| TNM stage |  |
| I | 5 |
| II | 13 |
| III | 12 |

Samples from 30 HCC patients were collected for flow cytometry analysis.
