# Supplementary material for: Downregulation of TPX2 impairs the antitumor activity of CD8+ T cells in hepatocellular carcinoma
Source: Cell Death Dis. 2022 Mar 10;13(3):223. doi: 10.1038/s41419-022-04645-8 (PMC8913637; doi:10.1038/s41419-022-04645-8)
Supplement: Supplementary file 7 — Supplementary Table S3 [file 41419_2022_4645_MOESM7_ESM.docx]

**Supplementary Table S3.** Characteristics of HCC patients’ samples used for RNA sequencing.

| ID | Sample | | HBV | Age  (years) | Gender | Tumor size (diameter/  cm) | Differen-tiation | Tumor capsular | TNM stage |
| --- | --- | --- | --- | --- | --- | --- | --- | --- | --- |
| 1 | | TIL-CD8 | Yes | 58 | male | 4.6 | Moderate | Complete | II |
| 3 | | TIL-CD8 | Yes | 64 | female | 5.5 | Well | Complete | II |
| 4 | | TIL-CD8 | No | 43 | male | 4.2 | Poorly | Incomplete | III |

*: TIL, tumor infiltrated lymphocytes.
