# Supplementary material for: Downregulation of TPX2 impairs the antitumor activity of CD8+ T cells in hepatocellular carcinoma
Source: Cell Death Dis. 2022 Mar 10;13(3):223. doi: 10.1038/s41419-022-04645-8 (PMC8913637; doi:10.1038/s41419-022-04645-8)
Supplement: Supplementary file 8 — Supplementary Table S4 [file 41419_2022_4645_MOESM8_ESM.docx]

**Supplementary Table S4. Regents used in this study.**

| **REAGENT** | **SOURCE** | **IDENTIFIER** |
| --- | --- | --- |
| **Antibodies** | | |
| Mouse anti-human PD-1 mAb, clone J116 | Abcam | Cat#ab140950 |
| PerCP-Cy5.5 mouse anti-Human CD8a, clone RPA-T8 | BioLegend | Cat#301032 |
| APC anti-human CD279 (PD-1), clone EH12.2H7 | BioLegend | Cat#329908 |
| PE-Cy7 anti-human CD366 (Tim-3), clone [F38-2E2](https://www.biolegend.com/en-us/search-results?Clone=F38-2E2) | BioLegend | Cat#345014 |
| Alexa Fluor 647 Mouse Anti-Human TIM-3 (CD366), clone 7D3 | BD Biosciences | Cat#565558 |
| Alexa Fluor 488 anti-human IFN-γ, clone 4S.B3 | BioLegend | Cat#502515 |
| PE mouse anti-human TNF, clone MAb11 | BD Biosciences | Cat#554513 |
| PE rabbit anti- Active Caspase-3, clone C92-605 | BD Biosciences | Cat#550821 |
| APC-Cy7 rat anti-mouse CD8a, clone 53-6.7 | BD Biosciences | Cat#557654 |
| PE Mouse Anti-Human CD45RA, Clone 5H9 | BD Biosciences | Cat#556627 |
| APC Mouse Anti-Human CD45RO, Clone UCHL1 | BD Biosciences | Cat#560899 |
| FITC Mouse Anti-Mouse CD45.1, Clone A20 | BD Biosciences | Cat#561871 |
| PE Mouse Anti-Mouse CD45.2, Clone 104 | BD Biosciences | Cat#560695 |
| FITC anti-mouse IFNg, clone XMG1.2 | eBioscience | Cat#11-7311-82 |
| PE-Cy7 rat anti-mouse TNF, clone MP6-XT22 | BD Biosciences | Cat#557644 |
| FITC anti-mouse Ki-67, clone 16A8 | BioLegend | Cat#652410 |
| Fixable Viability Stain 620 | BD Biosciences | Cat#564996 |
| Purified NA/LE mouse anti-human CD3, clone HIT3a | BD Biosciences | Cat#555336 |
| Purified NA/LE mouse anti-human CD28, clone CD28.2 | BD Biosciences | Cat#555725 |
| Rabbit anti-mouse CD8 alpha mAb, clone EPR21769 | Abcam | Cat#ab217344 |
| Anti-mouse IgG (H+L), F(ab')2 Fragment (APC Conjugate) | Cell Signaling Techonlogy | Cat#8887S |
| Rabbit TCF1/TCF7 mAb | Cell Signaling Techonlogy | Cat#2203S |
| NF-κB p65 (D14E12) XP® Rabbit mAb | Cell Signaling Techonlogy | Cat# 8242 |
| TPX2 (D2R5C) XP® Rabbit mAb | Cell Signaling Techonlogy | Cat#12245 |
| Anti-TPX2 antibody [EPR23180-4] | Abcam | Cat#ab252945 |
| Phospho-NF-κB p65 (Ser536) (93H1) Rabbit mAb | Cell Signaling Techonlogy | Cat#3033 |
| Anti-CXCR5 antibody [EPR23463-30] | Abcam | Cat#ab254415 |
| β-Actin Rabbit mAb | Cell Signaling Techonlogy | Cat#4970S |
| Alexa Fluor® 488 Rat Anti-Human CXCR5 (CD185) | BD Biosciences | Cat# 558112 |
| PerCP-Cy™5.5 Rat Anti-Human CXCR5 (CD185) | BD Biosciences | Cat# 562781 |
| Anti-mouse IgG (APC Conjugate) | Cell Signaling Techonlogy | Cat#4410S |
| Critical Commercial Reagent | | |
| RNeasy Mini Kit (50) | QIAGEN | Cat#74104 |
| Pierce™ Direct Magnetic IP/Co-IP Kit | Thermo Fisher Scientific | Cat#88828 |
| Ficoll-Paque PLUS | GE Healthcare | Cat#17-1440-02 |
| Ficoll-Paque PREMIUM 1.084 | GE Healthcare | Cat#17-5446-02 |
| Percoll | GE Healthcare | Cat#17-0891-09 |
| RBC Lysis Buffer (10X) | BioLegend | Cat#420301 |
| Flow Cytometry Staining Buffer (1X) | R&D | Cat#FC001 |
| Fixation/Permeabilization Solution Kit | BD Biosciences | Cat#554714 |
| FoxP3 Staining Buffer Set | Miltenyi | Cat#130-093-142 |
| Foxp3 / Transcription Factor Staining Buffer Set | eBioscience | Cat#00-5523-00 |
| EasySepTM human CD8+ T Cell Enrichment Kit | STEMCELL | Cat#19053 |
| EasySepTM human naive CD8+ T Cell Enrichment Kit | STEMCELL | Cat#19158 |
| EasySep™ Mouse CD90.2 Positive Selection Kit II | STEMCELL | Cat#18951 |
| EasySep™ Mouse T Cell Isolation Kit | STEMCELL | Cat#19851 |
| Dynabeads™ Human T-Activator CD3/CD28 for T Cell Expansion and Activation | Thermo Fisher Scientific | Cat#11131D |
| Recombinant Human IL-2 | PEPROTECH | Cat#200-02 |
| Recombinant Human IL-4 | PEPROTECH | Cat#200-04 |
| Recombinant Human GM-CSF | PEPROTECH | Cat#300-03 |
| Nivolumab | BMS | Cat#1365455 |
