# Supplementary material for: Downregulation of TPX2 impairs the antitumor activity of CD8+ T cells in hepatocellular carcinoma
Source: Cell Death Dis. 2022 Mar 10;13(3):223. doi: 10.1038/s41419-022-04645-8 (PMC8913637; doi:10.1038/s41419-022-04645-8)
Supplement: Supplementary file 9 — Supplementary Table S5 [file 41419_2022_4645_MOESM9_ESM.docx]

**Supplementary Table S5.** Primers used and siRNA for *TPX2* knockdown.

| Primer sequences | | |
| --- | --- | --- |
| Gene Symbol | **Forward Primer** | **Reverse Primer** |
| Human *TPX2* | TCCTGCCCGAGTGACTAAGG | CTGTTAGGGGTTCGTTTATGGAA |
| Human *β-actin* | CATGTACGTTGCTATCCAGGC | CTCCTTAATGTCACGCACGAT |
| Human *CXCR5* | CACGTTGCACCTTCTCCCAA | GGAATCCCGCCACATGGTAG |
| Mouse *Tpx2* | GATGCCCCCACCGACTTTATC | CTTGTTCTCCAAGTTGGCCTT |
| Mouse *β-actin* | GGCTGTATTCCCCTCCATCG | CCAGTTGGTAACAATGCCATGT |
| si-RNA sequences | | |
| Target Gene | **Targeting sequence** | |
| Human *TPX2* (si1) | GAAUGGAACUGGAGGGCUUTT | |
| Human *TPX2* (si2) | AUGAAAGUUUCUAACAACAAATT | |
| Mouse *Tpx2* (si) | TAATGATAGTGCATCCTCTGG | |
