# Supplementary material for: Downregulation of TPX2 impairs the antitumor activity of CD8+ T cells in hepatocellular carcinoma
Source: Cell Death Dis. 2022 Mar 10;13(3):223. doi: 10.1038/s41419-022-04645-8 (PMC8913637; doi:10.1038/s41419-022-04645-8)
Supplement: Supplementary file 10 — Information for review_ArrayExpress data [file 41419_2022_4645_MOESM10_ESM.doc]

ArrayExpress data

For reviewer access, please check:
http://www.ebi.ac.uk/arrayexpress/experiments/E-MTAB-10040

Username: Reviewer_E-MTAB-10040
Password: rmegffhj
